# Hearing impairment and risk of dementia in The HUNT Study (HUNT4 70+): a Norwegian cohort study

**DOI:** 10.1016/j.eclinm.2023.102319

**Published:** 2023-12-04

**Authors:** Christian Myrstad, Bo Lars Engdahl, Sergi Gonzales Costafreda, Steinar Krokstad, Frank Lin, Gill Livingston, Bjørn Heine Strand, Beate Øhre, Geir Selbæk

**Affiliations:** a The Norwegian National Centre for Ageing and Health, Vestfold Hospital Trust, Tønsberg, Norway; b Department of Medicine, Levanger Hospital, Nord-Trøndelag Hospital Trust, Levanger, Norway; c Department of Physical Health and Ageing, Norwegian Institute of Public Health, Oslo, Norway; d Division of Psychiatry, University College London, London, UK; e Faculty of Medicine and Health Sciences, Department of Public Health and Nursing, HUNT Research Centre, Norwegian University of Science and Technology, Trondheim, Norway; f Levanger Hospital, Nord-Trøndelag Hospital Trust, Levanger, Norway; g Cochlear Center for Hearing and Public Health, Johns Hopkins Bloomberg School of Public Health, Baltimore, Maryland, USA; h Department of Geriatric Medicine, Oslo University Hospital, Oslo, Norway; i The Norwegian National Unit for Sensory Loss and Mental Health, Oslo University Hospital, Norway; j Faculty of Medicine, Institute of Clinical Medicine, University of Oslo, Oslo, Norway; k Camden and Islington NHS Foundation Trust, London, UK

**Keywords:** Dementia, Alzheimer’s disease, Hearing impairment, Observational study, Cohort study

## Abstract

**Background:**

Hearing impairment is strongly associated with future dementia. No studies have reported objectively measured hearing impairment in a cohort with a long period of follow-up (>20 years), and few have reported follow-up over 10 years. Hence, there is a need for high quality studies with sufficient follow-up time and data to account for reverse causality and confounding. We aimed to address this knowledge gap.

**Methods:**

This cohort study used individual participant data from The Trøndelag Health Study (HUNT) in Norway. All current residents aged at least 20 years in the former Norwegian Nord-Trøndelag County were invited to participate in four decennial surveys: HUNT1 (1984–1986), HUNT2 (1995–1997), HUNT3 (2006–2008), and HUNT4 (2017–2019) with individuals aged at least 70 years included in a substudy, known as HUNT4 70+. Here, we report the findings of this substudy. HUNT4 70+ comprised 7135 participants who were assessed for dementia using the Diagnostic and Statistical Manual of Mental Disorders 5 criteria and who had audiometry between 1996 and 1998. The primary objective was to investigate, with gold standard audiometric testing and dementia diagnostic assessment, whether hearing impairment was an independent risk factor for all-cause dementia. The secondary objective was to investigate if a risk also applied to Alzheimer dementia and non-Alzheimer dementia. We analysed the association using Poisson regression and adjusted for confounders. This study is registered with ClinicalTrials.gov (NCT04284384).

**Findings:**

At baseline, 1058 (15%) individuals had acquired hearing impairment with a hearing threshold of at least 25 decibel (dB) and, at follow-up, 1089 (15%) had dementia. In the total group, people with hearing impairment had a relative risk (RR) 1.04 (95% confidence interval (CI) 1.00–1.09) per 10 dB increase in hearing thresholds. For individuals younger than 85 years at follow-up the RR was 1.12 (95% CI 1.05–1.21). Associations between hearing impairment and Alzheimer dementia and non-Alzheimer dementia were similar. There was no association for individuals aged at least 85 years.

**Interpretation:**

We found a moderate association between objectively measured hearing impairment and dementia in the younger age group (<85 years). The findings of no association in the older age group (≥85 years) might be due to the competing risk of death. The present study adds to the literature showing that acquired hearing impairment is a risk for dementias over a period which is too long for reverse causation, and with thorough consideration of confounders. Further research is needed to investigate associations between the different aetiologies of hearing loss and dementia subtypes, and risk differences for sexes.

**Funding:**

The Norwegian National Centre for Ageing and Health with a grant from Health South-East.

**Research in context**

**Evidence before this study**

The *Lancet* commission on dementia prevention, intervention and care in 2017, and the 2020 update, found that hearing impairment was the most important of the modifiable risk factors for dementia with a pooled relative risk (RR) of 1.94 (95% confidence interval [CI] 1.38–2.73), based on three studies. Lin and colleagues (2011) found a hazard ratio [HR] per 10 decibels hearing level [dB HL] of 1.27 (95% CI 1.06–1.50) in a cohort of 639 individuals with 58 incident cases of dementia. Gallacher and colleagues (2012) found an odds ratio per 10 dB HL of 2.67 (95% CI 1.38–5.18) in a cohort of 1057 men with 79 incident dementia cases. Deal and colleagues (2017) found a HR per 10 dB HL of 1.14 (95% CI 1.03–1.26) in a cohort of 1889 individuals with 229 incident dementia cases. After the latest *Lancet* commission report, Marinelli and colleagues (2022) found a HR per 10 dB HL of 0.99 (95% CI 0.89–1.07) in a cohort of 1159 individuals with 207 incident cases of dementia. A systematic review has not been published prior to this study. Hence, there is a need for high quality studies with sufficient follow-up time and data to account for reverse causality and confounding. We aimed to address this knowledge gap.

**Added value of this study**

We used data from a large cohort of 7135 individuals. Pure-tone air-conduction hearing thresholds were determined in sound-proof booths using a standardised automatic procedure, and manually when participants were unable to follow the instructions for the automatic procedure. Participants were followed over a mean of 21.7 years (range 20.3–23.2) making reverse causation unlikely, and reliable standardised methods to identify all-cause dementia and dementia subtypes at the endpoint assessment were used. With the longest follow-up on this topic so far, and with the best available evidence to date, this study found a RR of 1.04 (95% CI 1.00–1.09, p-value 0.054) with 1089 all-cause dementia cases. For individuals <85 years the RR was 1.12 (95% CI 1.05–1.21). The association with Alzheimer dementia and non-Alzheimer dementias was similar. We did not find an association for individuals ≥85 years. Higher mid-life comorbidities, lower educational levels and higher degrees of hearing impairment among dropouts may indicate that death acts as a competing risk to dementia and may be the reason for the lack of an association for the oldest participants. Our results show that the risk of future dementia for individuals with hearing impairment may appear excessively high if reverse causation or confounding variables are not taken into account.

**Implications of all the available evidence**

Present knowledge shows that hearing impairment is an individual risk factor for all-cause dementia, but probably with a lower magnitude than previously believed. Associations between hearing impairment and Alzheimer dementia and non-Alzheimer dementias in the literature vary in strength and between sexes. Whether hearing aid use decreases the risk is still uncertain, although recent observational studies indicate some benefit. Future study cohorts need to be dimensioned to investigate such associations, and new technology should be used in hearing aids to improve quality in studies on treatment effects.

## Introduction

In 2019, the estimated number of people with dementia was 57.4 million and is expected to increase to 152.8 million by 2050.^1^, ^2^, ^3^ The prevalence of hearing impairment was estimated at 64% for individuals >64.^4^ Twelve risk factors, which can be prevented or delayed, account for 40% of worldwide dementias.^5^ Among these, hearing impairment stands out as the most important potentially modifiable risk factor for dementia, and in 2017 the Lancet Commission identified in a meta-analysis a strong overall relative risk of 1.9 from studies of objective hearing loss in samples of mean ages 55–65 after 9–17 years follow-up. A study later divided them into risk per 10 dB of hearing loss, finding an odds ratio of 1.3.^6^^,^^7^ Yet there are concerns that this association is erroneous because hearing impairment causes people to be misdiagnosed as having dementia, and due to reverse causality. Hearing impairment may be an early symptom when people are developing dementia, as neurodegenerative illnesses often develop over a period of at least 10 years and up to 20 years prior to the appearance of clinical symptoms.^8^ There are few studies which report objective hearing impairment and the risk of dementia over 10 years follow-up and none over 20 years.

To our knowledge, only four high-quality studies exist, which applied the gold standard of audiometric testing and had at least five years of follow-up.^9^, ^10^, ^11^, ^12^ Lin and colleagues (2011) found an increasing risk for incident dementia with increase in severity of baseline hearing loss with a hazard ratio (HR) of 1.24 (95% confidence interval (CI) 1.04–1.48) per 10 decibel (dB) loss.^9^ The number of participants was 639, follow-up with a median of 11.9 years, mean age 63.7, and 58 (9%) had dementia. Gallacher and colleagues (2012) found an association between the mean hearing thresholds of two hearing assessments (in mean 8.6 years apart), and dementia risk with increase in hearing loss with odds ratio (OR) 2.67 (1.38–5.18) per 10 dB loss without adjustment for cardiovascular diseases.^10^ The participants included 1057 men, mean age 56.1, and 79 (7%) developed dementia. For some of these participants, diagnosis of dementia was obtained from medical records, and some auditory assessments were performed with background noise. The hearing loss was calculated as a mean of two assessments in wave one to four and therefore the length of follow-up is unclear, making their findings difficult to compare with other studies. Deal and colleagues (2017) found increased risk of incident dementia for individuals with an increase in severity of baseline hearing loss with HR 1.14 (1.03–1.25) per 10 dB. The 1889 participants were physically healthy septuagenarians (mean age 75.5) without difficulties performing activities of daily living (ADL), follow-up over 9 years, and 229 (12%) developed dementia.^11^ The dementia diagnoses were based on prescribed dementia medication, dementia diagnosis from hospital records or decline in the modified mini-mental state exam of more than 1.5 standard deviation (SD). Marinelli and colleagues (2022) found no association between objectively measured hearing threshold at baseline and dementia with HR 0.99 (0.89–1.12) per 10 dB. The number of participants was 1159, follow-up (7.0 years, SD 3.7), mean age 76.0, and 207 (18%) developed dementia.^12^

The primary objective of the present study was, with the best available evidence to date, to investigate whether hearing impairment was an independent risk factor for dementia. We have used the largest longitudinal study with gold standard audiometric testing and dementia diagnostic assessment. This research also includes the longest follow-up on this topic so far, with more than two decades, and adjustment for more possible confounding factors. The secondary objective was to investigate if a risk also applied to Alzheimer dementia (AD) and non-ADs.

## Methods

### Study design and participants

We used individual participant data from the Trøndelag Health Study (HUNT) in Norway.^13^ All current residents aged ≥20 years in the former Norwegian Nord-Trøndelag county were invited to participate in four decennial surveys: HUNT1 (1984–1986), HUNT2 (1995–1997), HUNT3 (2006–2008) and HUNT4 (2017–2019) with individuals ≥70 included in a substudy, HUNT4 70+. The county area is home to 140,000 people (2018) and the people differ little from the national average for cause-specific mortality, general health, unemployment rate and disability insurance, while the number of immigrants and proportion of people with higher educational levels are below the national average.^14^, ^15^, ^16^

**Fig. 1:**
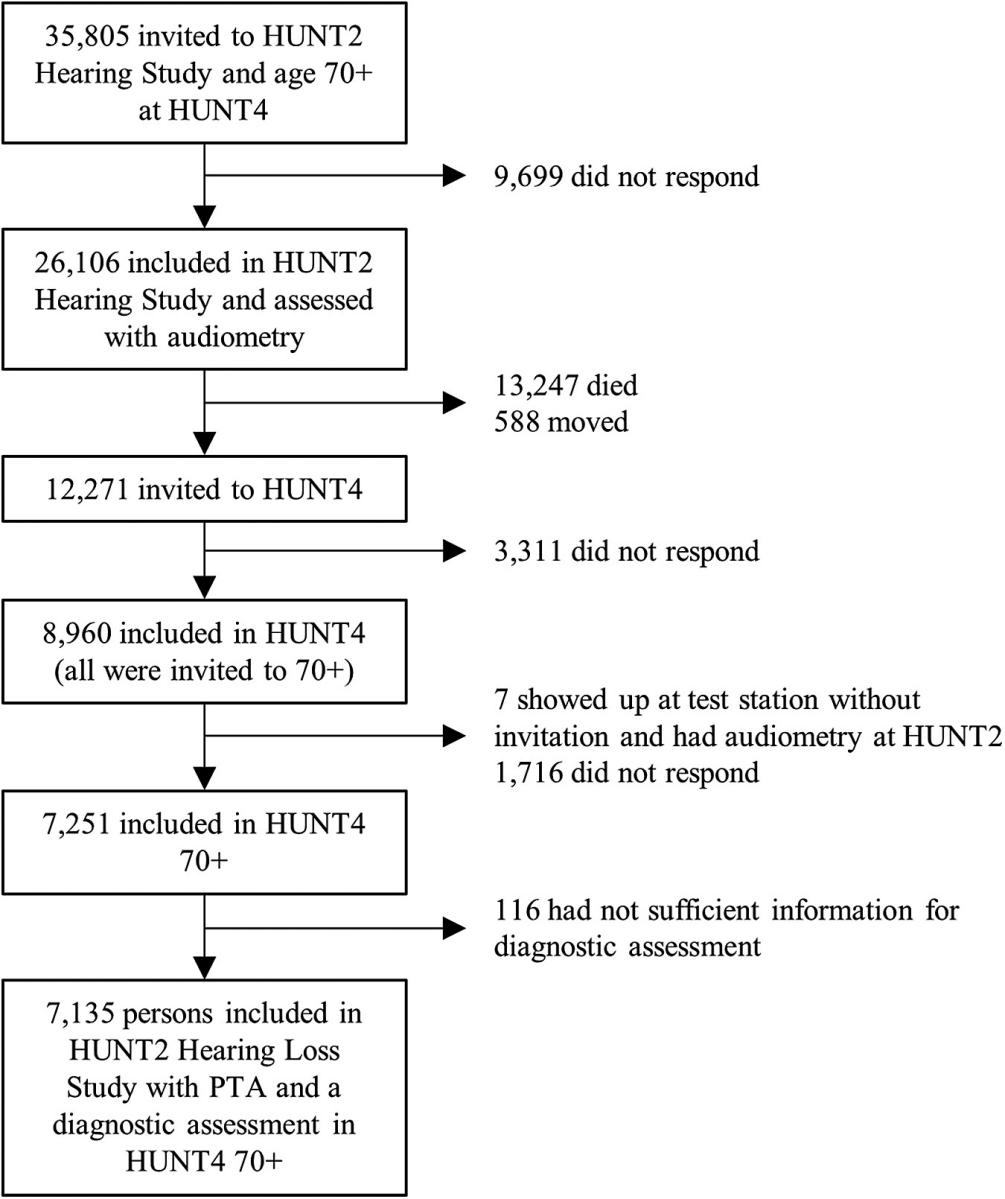
**Study profile.** Flow chart of study participants showing numbers and attrition in the Trøndelag Health Study, Norway.

Our study sample (N = 7135) comprised participants with hearing assessments in HUNT2 at ages 47–80 (mean 55.6, SD 5.9) and dementia assessment in HUNT4 70+ at ages 70–102 years (mean 78.2, SD 6.5). In the corresponding age group, in the HUNT2 Hearing Study (1996–1998) 26,106 (73% of invitees) participated and were included at baseline (Fig. 1).^16^^,^^17^

### Ethics

The Regional Committees for Medical and Health Research Ethics approved the study (‘23178 HUNT hørsel’). The full study protocol was registered with ClinicalTrials.gov (NCT04284384) and is available online.

### Exposures

The main exposure was the pure-tone average (hearing threshold) calculated as the average hearing thresholds of 0.5, 1, 2 and 4 kHz in the better-hearing ear measured in dB. We defined a hearing threshold <25 dB as normal hearing; 25–40 dB mild impairment (hearing aids usually recommended) and ≥40 dB as moderate or higher degree of impairment (hearing aids needed). Pure-tone air-conduction hearing thresholds were determined in sound-proof booths using an automatic procedure in accordance with ISO 8253-1, and manual audiometry was conducted with people unable to follow the instructions for the automatic procedure.^4^

A wide range of baseline variables associated with hearing impairment and dementia were included and used in different models. In Model A we adjusted for sex and age. To make the analysis comparable with previous research, we adjusted for education and cardiovascular risk factors diabetes mellitus, systolic blood pressure, smoking and stroke/bleeding in Model B.^9^, ^10^, ^11^ In the fully adjusted main analysis, Model C, we included all confounders, i.e. all confounders in Model B, hospitalisation due to head injury, body mass index (BMI), alcohol use, cholesterol and ischemic heart disease.^13^^,^^18^, ^19^, ^20^, ^21^, ^22^, ^23^, ^24^ Finally, to consider the impact of covariates that could potentially be on the causal pathway, we performed a sensitivity analysis in Model D as a further test of potential causal pathways (see Appendix 1). These were depression, physical activity, marital status and living alone.^18^^,^^20^^,^^25^

Directed acyclic graphs drawn with DAGitty, together with a priori knowledge and clinical judgements were used to map the possible causal associations between the variables.^26^ This graph and description of all variables can be found in the Appendixes 2 and 3.

### Outcomes

The outcomes of this study were all-cause dementia, Alzheimer dementia and non-ADs. The cognitive assessment protocol used to establish a diagnosis of dementia comprised a neurocognitive test battery, ADL, neuropsychiatric symptoms, subjective cognitive decline, first symptom with time of debut, symptom course and a caregiver interview.^27^, ^28^, ^29^ For each individual, two experts (and a third if no consensus was achieved) from a diagnostic work-up group of nine medical doctors with comprehensive scientific and clinical expertise, independently made diagnoses of dementia by applying the Diagnostic and Statistical Manual of Mental Disorders 5 using all available information.^30^^,^^31^

### Statistical analysis

Analyses were performed with Stata/SE 17.0 and 18.0. The relative risks (RR) of all-cause dementia, Alzheimer dementia, and non-ADs for hearing thresholds, and all p-values, were estimated using robust-error-variance in modified Poisson regression. We post-hoc chose to estimate risk ratios instead of odds ratios as odds ratios always overestimate risk ratios when outcomes are frequent. Poisson regression was chosen since it is a robust method to estimate risk ratios. To check for possible nonlinear relationships pure-tone average (PTA) was modelled post-hoc as restricted cubic spline with four knots. This did not create a better fit than the simpler model with PTA as a linear variable (Likelihoods-ratio test, p-value >0.05). As prespecified we first estimated the RR per 10 dB increase in hearing threshold, and then estimated the RR for dementia for those with hearing impairment vs. normal hearing (hearing threshold <25 dB). To assess whether the association depended on age and sex, we included all three- and two-way interactions between hearing threshold, age and sex (95% CI 0.98–0.99). In addition and as prespecified, we made analyses to assess selection bias. To investigate the effect of death as competing risk, the analyses were stratified by age at follow-up. The risk of loss to follow-up due to death is higher among the oldest and the cut-off was set to 85 years. As a post-hoc analysis after peer-review we also performed the analyses with cut-off 80 (See Appendix 1). The main analysis was run with a dataset where missing data (4-1,451 of 7135 cases) as prespecified was replaced with multiple imputation. As a prespecified sensitivity analysis, we subsequently ran the same analyses in a dataset without missing data (complete cases, n = 6186, 86.7%). For the post-hoc dropout analysis we estimated the risk of participating associated with the hearing threshold using Poisson regression cumulative odds model with adjustment for all confounders. We found no evidence of major violations of the proportional odds assumption, and ordinal logistic regression models are considered to be robust to violations.^32^ The significance level was set to 5% in all tests.

Out of the total study sample (N = 7135) none had missing data for outcome and exposure. A total of 75.4% had complete data for all covariates. Of those with missing covariates, 1.5% had three or more missing values.

Missing values for covariates were imputed using multiple imputation with chained equation, where all exposures and covariates in the main analysis were included in the procedure. In the imputation model, linear regression or predictive mean matching was performed for continuous, ordered logistic regression for ordinal and logistic regression for binary variables. To minimise bias, maximise use of available information and obtain appropriate estimates of uncertainty, 50 datasets were imputed. The distributions in histograms of the imputed values were visually compared to the observed ones to assess whether they were reasonable.^33^

### Role of the funding source

The funder did not have any role in study design, data collection, data analyses, interpretation, or writing of the report.

## Results

Between August 15, 1995 and June 18, 1997, 26,106 participants were enrolled. Of the 26,106 people at baseline 13,247 died before HUNT4 70+ took place, and 588 had moved outside the catchment area. Of the remaining 12,271 people, 7251 consented to participate, but 116 were excluded due to insufficient information. Thus, the final number of individuals with a valid hearing status in HUNT2 and assessment of cognitive status at HUNT4 was 7135 (see Fig. 1). Mean follow-up time was 21.7 years (SD 0.6, range 20.3–23.2). Baseline demographic characteristics of participants by hearing status category are presented in Table 1.

**Table 1:** Overview of the sample, prevalence of hearing impairment, demographic characteristics and covariates.

Baseline covariates	Hearing status^a^						Total		Missing (n)	Method^b^
	Normal		Mild impairment		Moderate/severe impairment					
Frequency (n, %)	6077	85.2	858	12.0	200	2.8	7135	100		
Dementia (n, %)	778	10.9	239	3.3	72	1.0	1089	15.3		
	**Mean**	**SD**	**Mean**	**SD**	**Mean**	**SD**	**Mean**	**SD**		
Age (years)	55.6	5.9	60.7	7.4	64.3	7.7	56.5	6.5	0	Calculated
Systolic blood pressure (mmHg)	138	19	143	20	145	20	139	19.4	21	Measured
BMI (kg/m^2^)	26.8	3.7	27.1	3.5	27.7	4.0	26.8	3.7	28	Measured
Cholesterol (mmol/l)	6.24	1.13	6.33	1.17	6.46	1.04	6.25	1.1	26	Measured
Physical activity index^c^	1.13	1.01	1.10	1.04	1.17	1.01	1.13	1.0	512	Self-reported
Alcohol use (times/month)	2.9	3.5	2.6	3.3	2.2	2.7	2.9	3.5	1451	Self-reported
HADS	7.5	5.1	7.6	4.9	7.6	4.9	7.5	5.1	786	Self-reported
	**n**	**%**	**n**	**%**	**n**	**%**	**n**	**%**		
Male sex	2578	42.4	499	58.2	115	57.5	3192	44.7	0	Self-reported
Living alone	1100	18.1	182	21.2	54	27.0	1336	18.7	0	Central register
Marital status	5027	82.7	687	80.1	150	75.0	5864	82.2	0	Central register
Education									0	Central register
Primary school	1447	23.8	291	33.9	75	37.5	1813	25.4		
Secondary school	3324	54.7	450	52.4	107	53.5	3881	54.4		
University, <4 years	1065	17.5	103	12.0	16	8.0	1184	16.6		
University, ≥4 years	241	4.0	14	1.6	2	1.0	257	3.6		
History of stroke/bleeding	39	0.6	10	1.2	5	2.5	54	0.8	5	Self-reported
Ischemic heart disease	85	1.4	29	3.4	7	3.5	121	1.7	6	Self-reported
Diabetes mellitus	119	2.0	26	3.0	5	2.5	150	2.1	4	Self-reported
Smoking status									71	Self-reported
Never	2748	45.2	373	43.5	87	43.5	3208	45.0		
Former	2047	33.7	332	38.7	74	37.0	2453	34.4		
Current	1223	20.1	142	16.6	38	19.0	1403	19.7		
Head injury	322	5.3	62	7.2	22	11.0	406	5.7	305	Self-reported

The HUNT Study, Norway.

SD = standard deviation; BMI = body mass index; HADS = Hospital Anxiety and Depression Scale.

a The main exposure was the pure-tone average (hearing threshold) calculated as the average pure-tone hearing thresholds of 0.5, 1, 2 and 4 kHz in the better-hearing ear measured in decibels (dB). A hearing threshold of less than 25 dB was defined as normal hearing; 25–40 dB mild impairment (hearing aids usually recommended); and greater than 40 dB was defined as moderate to severe and profound impairment (hearing aids needed).

b Data was collected from the Trøndelag Health Study 2 (HUNT2) questionnaires and measurements, measured by HUNT test personnel or collected from a central register (Statistics Norway).

c A continuous index for physical activity was calculated by a logarithm rewarding hours of vigorous activity per week over hours of low activity per week, as vigorous activity seems to be the form of movement with the most convincing dementia risk reduction.

Of all 7135 participants at baseline, 85.2% had normal hearing, 12.0% had mild hearing impairment, and 2.8% had moderate/severe hearing impairment. This is in line with the prevalence of hearing loss in all HUNT2 participants.^4^ Compared with participants without hearing impairment, those with hearing impairment were more likely to be older, male, have higher BMI, higher blood pressure, higher cholesterol, a history of stroke/bleeding, ischemic heart disease and hospitalisation caused by a head injury. They were also less often married, more often living alone, had lower educational levels, less frequent alcohol consumption and smoked less (see Table 1). At follow-up, the all-cause dementia prevalence was 15.3%. The prevalence of Alzheimer dementia was 8.7% and non-ADs 6.6%.

The crude association between hearing impairment and dementia per 10 dB increase in hearing threshold was RR 1.38 (95% CI 1.33–1.44), for women RR 1.43 (95% CI 1.36–1.51) and for men RR 1.36 (95% CI 1.27–1.46). In the main fully adjusted analysis (Model C), there was a small association between hearing impairment and dementia with RR 1.04 (95% CI 1.00–1.09); however, there was a clear association in those <85 with RR 1.12 (95% CI 1.05–1.21). In <85 the association remained when analysed separately for men, RR 1.12 (95% CI 1.01–1.23) and women RR 1.15 (95% CI 1.03–1.27) (See Table 2 and Forest plot in Appendix 6). For all models A–C, when stratified by age and sex, there were associations for individuals <85 at follow-up but not ≥85, and associations for men and women <85. There was a small two-way interaction between increase in hearing threshold and age only in Model C (95% CI 0.98–0.99, p < 0.001). There was an increased risk for Alzheimer dementia in those <85, and in the total group of men and for men <85. There was an increased risk for non-AD in women <85 (See Table 3).

**Table 2:** Relative risk (RR) for all-cause dementia per 10 decibels increase in hearing threshold in the whole sample, men and women separately and stratified by age at follow-up (n = 7135).

Age, years	Participants	Dementia cases	Model A			Model B			Model C		
	n	n	RR	95% CI	p value	RR	95% CI	p value	RR	95% CI	p value
Total	7135	1089	1.07	1.02–1.12	0.003	1.05	1.00–1.09	0.044	1.04	1.00–1.09	0.054
<85	5956	565	1.16	1.08–1.24	<0.001	1.12	1.05–1.20	0.001	1.12	1.05–1.21	0.001
≥85	1179	524	1.02	0.97–1.07	0.43	1.02	0.97–1.07	0.65	1.01	0.96–1.07	0.60
Women	3943	654	1.05	0.99–1.11	0.12	1.03	0.97–1.09	0.30	1.03	0.97–1.09	0.31
<85	3184	295	1.18	1.06–1.30	0.002	1.15	1.04–1.27	0.008	1.15	1.03–1.27	0.010
≥85	759	359	1.02	0.96–1.08	0.52	1.01	0.95–1.08	0.70	1.01	0.95–1.07	0.79
Men	3192	435	1.11	1.04–1.19	0.003	1.07	1.00–1.15	0.063	1.06	0.99–1.14	0.079
<85	2772	270	1.15	1.05–1.26	0.003	1.11	1.01–1.22	0.026	1.12	1.01–1.23	0.028
≥85	420	165	1.06	0.97–1.17	0.21	1.01	0.92–1.11	0.83	1.01	0.92–1.11	0.76

Model A: Adjustment for age and sex; Model B: Additional adjustment for education, diabetes mellitus, systolic blood pressure, smoking and stroke/bleeding; Model C (main analysis): Additional adjustment for head injury, BMI, alcohol use, cholesterol, and ischemic heart disease.

The RR, CI, and all p-values were estimated using robust-error-variance in modified Poisson regression.

RR = relative risk; CI = confidence interval; BMI = body mass index.

**Table 3:** Relative risk for dementia subtypes per 10 decibels increase in hearing threshold in the whole sample, men and women separately and stratified by age at follow-up.

Age, years	Participants	Dementia cases	AD cases			Participants	Dementia cases	Non-AD cases		
	n	n	RR	95% CI	p value	n	n	RR	95% CI	p value
Total	6666	620	1.05	0.99–1.12	0.091	6515	469	1.05	0.97–1.13	0.20
<85	5703	312	1.15	1.05–1.26	0.003	5644	253	1.10	0.99–1.24	0.089
≥85	963	308	1.01	0.94–1.09	0.79	871	216	1.03	0.93–1.13	0.59
Women	3672	383	1.02	0.95–1.10	0.60	3560	271	1.06	0.95–1.17	0.28
<85	3053	164	1.13	0.98–1.30	0.088	3020	131	1.19	1.02–1.38	0.027
≥85	619	219	1.00	0.92–1.09	0.98	540	140	1.01	0.90–1.14	0.83
Men	2994	237	1.10	1.00–1.22	0.047	2955	198	1.04	0.93–1.17	0.49
<85	2650	148	1.17	1.03–1.32	0.012	2624	122	1.05	0.88–1.24	0.61
≥85	344	89	1.02	0.88–1.19	0.78	331	76	1.04	0.88–1.23	0.64

Model C with adjustment for age, sex, education, diabetes mellitus, systolic blood pressure, smoking, stroke/bleeding, head injury, body mass index, alcohol use, cholesterol and ischemic heart disease. Relative risk (RR) for AD and non-ADs (vascular dementia, Lewy-body dementia/Parkinson dementia, frontotemporal dementia, mixed dementia, other specified dementia, unspecified dementia) per 10 decibels increase in hearing threshold stratified by sex and age.

Participants with non-ADs are left out of the analysis of Alzheimer dementia-risk and vice versa.

The RR, CI, and all p-values were estimated using robust-error-variance in modified Poisson regression.

CI = confidence interval; AD = Alzheimer dementia.

We analysed RR for all-cause dementia, Alzheimer dementia and non-ADs in those with normal hearing vs. those with hearing impairment (>25 dB). In the total group there was a small association with all-cause dementia. There was an increased risk in those <85 and in women <85. In those <85 there was also an increased risk for Alzheimer dementia and non-ADs. There was an increased risk for non-ADs in women <85 (See Table 4).

**Table 4:** Relative risk for dementia with normal hearing vs. hearing impairment (≥25 dB) in the whole sample, men and women separately and stratified by age at follow-up.

Age, years	All-cause dementia (n = 7135)			AD cases (n = 6666)			Non-AD cases (n = 6515)		
	RR	95% CI	p value	RR	95% CI	p value	RR	95% CI	p value
Total	1.09	0.97–1.24	0.15	1.06	0.89–1.26	0.50	1.17	0.94–1.44	0.16
<85	1.36	1.11–1.67	0.003	1.34	1.01–1.78	0.044	1.46	1.05–2.03	0.024
≥85	1.02	0.89–1.17	0.76	0.99	0.82–1.20	0.95	1.06	0.83–1.35	0.66
Women	1.08	0.92–1.27	0.32	1.01	0.81–1.26	0.12	1.22	0.91–1.64	0.19
<85	1.58	1.17–2.14	0.003	1.35	0.85–2.13	0.20	2.05	1.31–3.22	0.002
≥85	1.02	0.87–1.20	0.79	1.01	0.81–1.27	0.92	1.02	0.73–1.41	0.92
Men	1.09	0.90–1.32	0.35	1.12	0.85–1.47	0.41	1.09	0.81–1.48	0.57
<85	1.24	0.94–1.62	0.13	1.36	0.95–1.95	0.094	1.11	0.70–1.75	0.66
≥85	0.98	0.77–1.24	0.86	0.93	0.65–1.33	0.68	1.07	0.72–1.58	0.92

Model C with adjustment for age, sex, education, diabetes mellitus, systolic blood pressure, smoking, stroke/bleeding, head injury, body mass index, alcohol use, cholesterol, and ischemic heart disease.

Relative risk (RR) for normal hearing vs. hearing impairment (≥25 decibels) stratified by sex and age.

The RR, CI, and all p-values were estimated using robust-error-variance in modified Poisson regression.

CI = confidence interval; AD = Alzheimer dementia; non-AD = vascular dementia, Lewy-body dementia/Parkinson dementia, frontotemporal dementia, mixed dementia, other specified dementia, unspecified dementia.

In the complete case analysis (n = 6186), there were no substantial changes in the associations compared with analysis after multiple imputation (see Appendix 4). The associations when adjusting for additional covariates (Model D) were similar to the association when adjusting only for confounders (Model C) but had wider CI’s (see Appendix 1).

As a drop-out analysis, and to investigate the difference in risk between the two age groups, we estimated the risk of participating in the study associated with the hearing threshold. Dropouts had higher mid-life comorbidities, lower educational levels and higher degrees of hearing impairment (hearing threshold +1.1 dB, p < 0.001 after controlling for age and sex).

## Discussion

The aim of the current study was to investigate the association between hearing impairment and future dementia in a sample from a large population-based cohort with a lengthy follow-up. This makes reverse causation unlikely. After adjusting for multiple demographic characteristics and known confounders, in the whole group the association between hearing impairment and dementia was RR 1.04 per 10 dB loss in hearing threshold at baseline, which had wide confidence parameters. However, when stratified by age there was a moderate 12% increased risk associated with 10 dB hearing loss in those under age 85. In the total group, with hearing impairment as a bivariate exposure, the association between hearing impairment and dementia was RR 1.09, which had wide confidence parameters as well, but when stratified by age there was a 36% increased risk in those under age 85. In the dementia subgroup analyses there were moderate associations with Alzheimer dementia for men in the total group with 17% increased risk and with non-ADs for women <85 years with 19% increased risk.

Our results support earlier findings of an association between hearing impairment and increased risk of dementia. The magnitude of the association is comparable with earlier findings in longitudinal studies of audiometric hearing impairment and dementia with confidence intervals that overlap ours.^9^^,^^11^^,^^12^ There may be a missed opportunity to diagnose dementia before dropout due to death in a long follow-up cohort prevalence study, as people with dementia tend to die earlier than their peers. This, together with an increase with age in the prevalence of confounding factors, might weaken the association among the oldest participants and may be a reason for the absence of an association in individuals ≥85 in our study.

Lin (2011), Gallacher (2012) and Deal and colleagues (2017) all found a larger increase in risk with baseline hearing loss and all-cause dementia than in our study. Marinelli and colleagues (2022) found no association. Still, except for the study by Gallacher and colleagues, which used a different methodology, these studies all have CIs that overlap with ours. Lin and colleagues studied participants who were younger at follow-up in a sample with a lower dementia prevalence, while Deal’s participants, all in their seventies, had an almost doubled dementia prevalence compared with septuagenarians in our study. Marinelli and colleagues found no association between hearing threshold at baseline and dementia in a population-based sample, with the same age at follow-up as in our study and the analysis with adjustment for most known confounders. All had a shorter time of follow-up.

Hearing impairment and dementia are associated with common risk factors. Although the association remained, it weakened along with more adjustments for such confounders. Compared with those who dropped out, those who participated in this study had better hearing, less mid-life comorbidities and higher education level (see Appendix 5). These factors may attenuate the association between hearing impairment and dementia in our study, which is supported by our results that show an association among the <85 age group but not among the ≥85 age group, and indicate that death might act as a competing risk to dementia.

In subgroup analyses, there was an increased risk of Alzheimer dementia for men, but only a weak association for women, despite the often-reported higher frequency of AD for women.^34^ In non-ADs, we found an association for women <85 but no association for men. The associations for dementia subtypes may be explained by lack of power due to few dementia-subtype cases.

In the current study, the increased risk for all-cause dementia per 10 dB hearing loss for individuals <85 years spans between 11% and 18% depending on model, age, and sex. This confirms to a large extent what we have seen in previous high-quality research, i.e. that acquired hearing impairment is an independent risk factor for dementia. Our results challenge the idea that hearing loss is associated with increased risk of cognitive impairment and dementia only in those with severe loss, showing a moderate increase in risk of dementia with only ten decibels increase in hearing threshold.^35^

The strengths of our study include a gold standard identification of both exposure with pure tone audiometry, and outcomes developed through an expert review of cognitive tests. Cognitive diagnoses were based on standardised criteria, and assessments were consistent throughout the whole sample, including a consensus process using a clinical expert panel.^31^ This is a strength compared to studies relying on less rigorous diagnoses, such as those from hospital admissions or from death records. No previous high-quality studies have reported follow-up over 20 years, and few over 10 years. We did not measure dementia at baseline, but with this length of follow-up, and the usual survival time with dementia, it is unlikely that any participants had dementia when hearing impairment was initially measured, and consequently, the risk of reverse causality is minimised.^36^ Further, the large sample size and a substantial number of confounders measured has allowed for extensive adjustments and sensitivity analyses. However, people with illnesses including dementia tend to differentially be lost to follow-up and this would be expected to reduce our associations.

There are limitations as well. In a geriatric population with considerable comorbidities, the competing risk of death is especially high, as previously discussed, and this may cause an underestimation of the true association. Alzheimer biomarkers were not collected and brain imaging was not performed, which may contribute to misclassification of dementia subtypes. Assessment of central hearing loss in addition to peripheral hearing loss should be considered in future studies of the association with risk of dementia. Even though our study is the largest performed so far, it may be underpowered to identify weaker associations between hearing impairment and dementia. With a one-sided test with 80% power and significance of 5%, our data had the potential to detect a minimum effect size of RR = 1.28.

The causal associations between the variables were mapped with directed acyclic graphs. Still, with an observational design, the ability to investigate a causal pathway between hearing impairment and dementia is limited. Nevertheless, theories point to a causal association, like effects of long-term deprivation of auditory input with psychosocial consequences and accelerated brain atrophy, and deprivation of cognitive reserves, because more resources must be devoted to process sound.^37^, ^38^, ^39^ Due to the observational study design, we do not know when the conditions began. Hence, mediating effects are difficult to differentiate from confounding effects. Further, we cannot rule out bias due to unmeasured confounders and residual confounding due to measurement errors in the confounders such as smoking, physical activity and alcohol consumption. However, the association between these known risk factors for dementia and hearing loss is found to be very weak and controlling for these confounders had a marginal effect on the estimates. As for the confounders, the associations remained when adjusting for the potentially causal factors depression, marital status, living alone and physical activity in the analysis.

Acquired hearing impairment was associated with an increased risk of all-cause dementia for people under age 85. For people aged 85 years and older there were no associations, possibly due to survival bias. Our study shows that acquired hearing impairment is a risk factor for dementia independent of comorbidity, sociodemographic and lifestyle factors. We have added to the literature with our long follow-up and extensive adjustment for confounding that the risk for future dementia for individuals with acquired hearing loss may appear too strong if we do not account for reverse causation or confounding variables.

Further research is necessary to investigate which types of hearing loss influence cognitive decline, which non-Alzheimer dementia subtypes are associated with hearing impairment, whether both sexes are at risk for all-cause dementia or dementia subtypes, and whether treatment for hearing loss could reduce the incidence of dementia.

As both conditions impact heavily on public health, even small effect sizes make an association important. Precise measures to reduce these conditions are of great importance to reduce societal load and improve quality of life.

## Contributors

CM: Conceptualisation, resources, data curation, project administration, formal analysis, validation, access and verification of the underlying data, investigation, visualisation, methodology, writing of original draft and editing. BLE: Conceptualization, resources, data curation, formal analysis, supervision, validation, access and verification of the underlying data, investigation, methodology, writing review and editing. SGC: Supervision, methodology, writing, reviewing and editing. SK: Planning, design, collaboration and implementation of data collection, writing, reviewing and editing. FL: Writing, reviewing and editing. GL: Supervision, methodology, writing, reviewing and editing. BHS: Data curation, formal analysis, supervision, validation, methodology, writing, reviewing and editing. BØ: Writing, reviewing and editing. GS: Conceptualisation, resources, project administration, formal analysis, supervision, funding acquisition, validation, investigation, methodology, writing, reviewing and editing.

## Data sharing statement

The data for this study encompasses information on the health and samples from participants in The Nord-Trøndelag Health Study. Researchers can access the data by application to the Regional Committees for Medical Health Research Ethics and the data owners (HUNT and Statistics Norway). The authors cannot share these data. However, other researchers may contact the authors if they have questions concerning the data.

## Declaration of interests

SGC has in the last 36 months received grants for research in dementia prevention, including by treating hearing loss, from UK NIHR, grant for dementia risk assessment from Dunhill Medical Trust—UK Charity, and grant for research in dementia prevention, including by treating hearing loss, from Alzheimer’s Research UK—Charity. FL has in the last 36 months received research grants pertaining to hearing loss from National Institutes of Health, research grants pertaining to hearing loss from Eleanor Schwartz Charitable Foundation, consulting fees as consultant on topics related to hearing loss from Frequency Therapeutics and Apple Inc., personal fees as expert witness for the plaintiff in a class action lawsuit against an insurance company in Washington state pertaining to litigation relating to the insurance company’s policy of non-coverage for hearing aids, is a scientific advisory board member for Fondation Pour L’Audition, is a scientific advisory board member (possible stock options pending continued role on the SAB), is a volunteer board of the nonprofit AccessHears, received donation in-kind from Sonova to Johns Hopkins University for hearing technologies used in the NIH-funded ACHIEVE trial, and is director of a public health research center funded in part by a philanthropic donation from Cochlear Ltd., to the Johns Hopkins Bloomberg School of Public Health. GL has since the initial planning of the work received payment made to the institutions University College London Hospitals’ National Institute for Health Research (NIHR), and Biomedical Research Centre, North Thames NIHR Applied Research Collaboration, and as an NIHR Senior Investigator to support academics to work. GS has participated in advisory boards for Biogen, Eisai and Roche concerning antidementia drugs. All other authors declare no competing interests.

## Figures and Tables

**Appendix 2:**
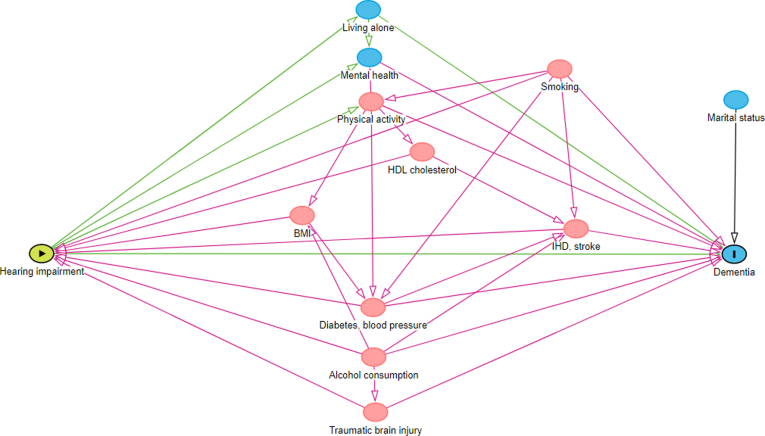
Directed acyclic graphs drawn with DAGitty.

## Appendix A Supplementary data

Appendix 6

Translated Abstract
