# Supplementary material for: Hearing impairment and risk of dementia in The HUNT Study (HUNT4 70+): a Norwegian cohort study
Source: eClinicalMedicine. 2023 Dec 4;66:102319. doi: 10.1016/j.eclinm.2023.102319 (PMC10772264; doi:10.1016/j.eclinm.2023.102319)
Supplement: Appendix 1 [file mmc1.docx]

Appendix 1. Extended Table 2 – Relative risk for all-cause dementia per 10dB increase in hearing loss in the whole sample, men and women separately and stratified by age

|  | **Age** | **Participants** | **Dementia cases** | **Model A** | | | **Model B** | | | **Model C** | | | **Model D** | | |
| --- | --- | --- | --- | --- | --- | --- | --- | --- | --- | --- | --- | --- | --- | --- | --- |
|  | **Years** | **n** | **n** | **RR** | **95% CI** | **P Value** | **RR** | **95% CI** | **P Value** | **RR** | **95% CI** | **P Value** | **RR** | **95% CI** | **P Value** |
| **Total** |  | 7135 | 1089 | 1·07 | 1·02-1·12 | 0·003 | 1·05 | 1·00-1·09 | 0·044 | 1·04 | 1·00-1·09 | 0·054 | 1·04 | 0·99-1·09 | 0·091 |
|  | <85 | 5956 | 565 | 1·16 | 1·08-1·24 | <0·001 | 1·12 | 1·05-1·20 | 0·001 | 1·12 | 1·05-1·21 | 0·001 | 1·11 | 1·03-1·19 | 0·004 |
|  | ≥85 | 1179 | 524 | 1·02 | 0·97-1·07 | 0·43 | 1·02 | 0·97-1·07 | 0·52 | 1·01 | 0·96-1·07 | 0·60 | 1·01 | 0·96-1·07 | 0·67 |
|  | <80 | 4761 |  |  |  |  |  |  |  | 1·15 | 1·05-1·26 | 0·002 |  |  |  |
|  | ≥80 | 2374 |  |  |  |  |  |  |  | 1·03 | 0·98-1·08 | 0·29 |  |  |  |
| **Women** |  | 3943 | 654 | 1·05 | 0·99-1·11 | 0·12 | 1·03 | 0·97-1·09 | 0·30 | 1·03 | 0·97-1·09 | 0·31 | 1·03 | 0·97-1·09 | 0·38 |
|  | <85 | 3184 | 295 | 1·18 | 1·06-1·30 | 0·002 | 1·15 | 1·04-1·27 | 0·008 | 1·15 | 1·03-1·27 | 0·010 | 1·13 | 1·02-1·26 | 0·017 |
|  | ≥85 | 759 | 359 | 1·02 | 0·96-1·08 | 0·52 | 1·01 | 0·95-1·08 | 0·70 | 1·01 | 0·95-1·07 | 0·79 | 1·01 | 0·95-1·07 | 0·80 |
|  | <80 | 2520 |  |  |  |  |  |  |  | 1·23 | 1·07-1·41 | 0·003 |  |  |  |
|  | ≥80 | 1423 |  |  |  |  |  |  |  | 1·01 | 0·95-1·07 | 0·76 |  |  |  |
| **Men** |  | 3192 | 435 | 1·11 | 1·04-1·19 | 0·003 | 1·07 | 1·00-1·15 | 0·063 | 1·06 | 0·99-1·14 | 0·079 | 1·06 | 0·99-1·13 | 0·11 |
|  | <85 | 2772 | 270 | 1·15 | 1·05-1·26 | 0·003 | 1·11 | 1·01-1·22 | 0·026 | 1·12 | 1·01-1·23 | 0·028 | 1·10 | 1·00-1·22 | 0·052 |
|  | ≥85 | 420 | 165 | 1·06 | 0·97-1.17 | 0·21 | 1·01 | 0·92-1·11 | 0·83 | 1·01 | 0·92-1·11 | 0·76 | 1·01 | 0·92-1·12 | 0·76 |
|  | <80 | 2241 |  |  |  |  |  |  |  | 1·11 | 0·98-1·25 | 0·94 |  |  |  |
|  | ≥80 | 951 |  |  |  |  |  |  |  | 1·05 | 0·97-1·15 | 0·24 |  |  |  |
