# Supplementary material for: Hearing impairment and risk of dementia in The HUNT Study (HUNT4 70+): a Norwegian cohort study
Source: eClinicalMedicine. 2023 Dec 4;66:102319. doi: 10.1016/j.eclinm.2023.102319 (PMC10772264; doi:10.1016/j.eclinm.2023.102319)
Supplement: Appendix 3 [file mmc2.docx]

**Appendix 3. Covariates**

**Alcohol use** was divided in four ordinal groups based on times of use per month, from never, to 1-7, 8-18 or 19-31 per month. Units of alcohol is not counted for.

A continuous index for **physical activity** was calculated by a logarithm rewarding hours of vigorous activity per week over hours of low activity per week, as vigorous activity seems to be the form of activity with significant influence on dementia risk. A low score indicates inactivity, and the method is found to correlate with morbidity in HUNT.

A **history of stroke/bleeding**, **ischemic heart disease**, **hospitalisation because of traumatic brain injury**, and **diabetes mellitus** was considered prevalent if answered “yes” in “Have you ever had”-questions.

**Blood pressure** was measured (mmHg) three times, and the mean of the second and third measurement was used as a continuous variable.

**Smoking** was grouped in never, former and current smoking, as the evidence is growing for current smoking as a stronger risk than former smoking.

**Body Mass Index** (BMI) as an assessment of obesity, and serum **HDL cholesterol** (mmol/dl), was measured on continuous scales.

**Hospital Anxiety and Depression Scale** (HADS) is used as indicator for mental health. As the variable has numerous missing items, answers in three other tools for assessment of mental health in HUNT2 were included when performing multiple imputation. Those were the Symptom Check List 10 (SCL-10), The Connor Mental Health Index (MHI), and The Four-Item Anxiety and Depression Index (ADI-4) with questions partly overlapping the questions in HADS.

**Marital status** (married/not married) and **educational level** (unspecified/none, primary school, secondary school, less than four years university, four or more years university) are collected from connected register Statistics Norway (SSB).

**Living alone** is used as proxy for social isolation and found by the number of persons living on the same address (SSB).
