# Supplementary material for: Hearing impairment and risk of dementia in The HUNT Study (HUNT4 70+): a Norwegian cohort study
Source: eClinicalMedicine. 2023 Dec 4;66:102319. doi: 10.1016/j.eclinm.2023.102319 (PMC10772264; doi:10.1016/j.eclinm.2023.102319)
Supplement: Appendix 4 [file mmc3.docx]

Appendix 4. Relative risk for all-cause dementia per 10dB increase in hearing loss for complete cases, men and women separately and stratified by age

|  | **Age** | **Partici-pants** | **Model A** | | | **Partici-pants** | **Model B** | | | **Partici-pants** | **Model C** | | |
| --- | --- | --- | --- | --- | --- | --- | --- | --- | --- | --- | --- | --- | --- |
|  | **Years** | **n** | **RR** | **95% CI** | **P Value** | **n** | **RR** | **95% CI** | **P Value** | **n** | **RR** | **95% CI** | **P Value** |
| **Total** |  | 7135 | 1·07 | 1·01-1·13 | 0·015 | 7053 | 1·05 | 1·00-1·10 | 0·046 | 6186 | 1·06 | 1·01-1·11 | 0·026 |
|  | <85 | 5956 | 1·16 | 1·07-1·25 | <0·001 | 5898 | 1·13 | 1·05-1·21 | 0·001 | 5221 | 1·12 | 1·04-1·21 | 0·002 |
|  | ≥85 | 1179 | 1·02 | 0·95-1·10 | 0·58 | 1155 | 1·02 | 0·96-1·07 | 0·57 | 965 | 1·03 | 0·97-1·09 | 0·38 |
| **Women** |  | 3943 | 1·05 | 0·97-1·13 | 0·23 | 3892 | 1·03 | 0·97-1·09 | 0·36 | 3318 | 1·05 | 0·98-1·12 | 0·15 |
|  | <85 | 3184 | 1·18 | 1·05-1·32 | 0·006 | 3149 | 1·15 | 1·04-1·27 | 0·008 | 2716 | 1·15 | 1·03-1·28 | 0·010 |
|  | ≥85 | 759 | 1·02 | 0·93-1·12 | 0·67 | 743 | 1·01 | 0·94-1·07 | 0·87 | 602 | 1·03 | 0·96-1·11 | 0·39 |
| **Men** |  | 3192 | 1·11 | 1·02-1·20 | 0·013 | 3161 | 1·07 | 1·00-1·15 | 0·049 | 2868 | 1·06 | 0·99-1·14 | 0·11 |
|  | <85 | 2772 | 1·15 | 1·04-1·28 | 0·008 | 2749 | 1·12 | 1·02-1·23 | 0·022 | 2505 | 1·11 | 1·00-1·23 | 0·044 |
|  | ≥85 | 420 | 1·06 | 0·94-1.20 | 0·34 | 412 | 1·02 | 0·93-1·12 | 0·72 | 363 | 1·02 | 0·92-1·12 | 0·76 |
