# Supplementary material for: Hearing impairment and risk of dementia in The HUNT Study (HUNT4 70+): a Norwegian cohort study
Source: eClinicalMedicine. 2023 Dec 4;66:102319. doi: 10.1016/j.eclinm.2023.102319 (PMC10772264; doi:10.1016/j.eclinm.2023.102319)
Supplement: Appendix 5 [file mmc4.docx]

Appendix 5· Risk of drop-out from baseline to follow-up due to mid-life comorbidities

| **Baseline covariates** | **Odds ratio** | **P Value** | **95% Confidence Interval** |
| --- | --- | --- | --- |
| Hearing threshold (per dB) | 0·99 | <0·001 | 0·99-1·00 |
| Age (year) | 0·91 | <0·001 | 0·90-0·91 |
| Male gender | 0·83 | <0·001 | 0·77-0·90 |
| Diabetes mellitus (yes/no) | 0·70 | 0·002 | 0·55-0·88 |
| Systolic blood pressure (mmHg) | 0·99 | <0·001 | 0·99-0·99 |
| Former smoker (yes/no) | 0·85 | <0·001 | 0·78-0·93 |
| Current smoker (yes/no) | 0·39 | <0·001 | 0·35-0·43 |
| Head injury, don’t remember | 0·98 | 0·79 | 0·83-1·15 |
| Head injury, hospitalization | 0·53 | 0·025 | 0·31-0·92 |
| Secondary school | 1·66 | <0·001 | 1·52-1·80 |
| University, <4years | 2·12 | <0·001 | 1·87-2·41 |
| University, >=4 years | 1·79 | <0·001 | 1·44-2·22 |
| BMI (kg/m2) | 0·98 | 0·002 | 0·97-0·99 |
| Alcohol use | 1·02 | 0·004 | 1·01-1·03 |
| Cholesterol (mmol/l) | 1·02 | 0·18 | 0·99-1·06 |
| Ischemic heart disease (yes/no) | 0·46 | <0·001 | 0·36-0·58 |
| Stroke/bleeding (yes/no) | 0·33 | <0·001 | 0·22-0·49 |
