# Supplementary material for: Hearing impairment and risk of dementia in The HUNT Study (HUNT4 70+): a Norwegian cohort study
Source: eClinicalMedicine. 2023 Dec 4;66:102319. doi: 10.1016/j.eclinm.2023.102319 (PMC10772264; doi:10.1016/j.eclinm.2023.102319)
Supplement: Translated Abstract [file mmc6.docx]

*The following translations in Norwegian were submitted by the authors and we reproduce them as supplied. They have not been peer reviewed. Our editorial processes have only been applied to the original abstract in English, which should serve as reference for this manuscript.*

**Sammendrag**

**Bakgrunn** Redusert hørsel er sterkt assosiert med fremtidig demenssykdom. Ingen studier har rapportert objektivt målt hørselssvekkelse i en kohort med lang oppfølgingstid (>20 år) og få med oppfølgingstid over 10 år. Det er derfor behov for studier med høy kvalitet, med tilstrekkelig oppfølgingstid og datagrunnlag for å redegjøre for revers kausalitet og konfundering.

**Metode** Deltakerne i denne studien er fra Helseundersøkelsen i Trøndelag (HUNT). Alle innbyggere 20 år eller eldre i tidligere Nord-Trøndelag fylke ble invitert til å delta i fire undersøkelser med 11 års mellomrom: HUNT1 (1984–1986), HUNT2 (1995–1997), HUNT3 (2006–2008) og HUNT4 (2017–2019), og med deltakere som var 70 år eller eldre i en egen delstudie kalt HUNT4 70+. Her rapporterer vi resultater fra denne delstudien. HUNT4 70+ inkluderte 7.135 deltakere som ble utredet for demens og diagnostisert etter kriteriene i Diagnostic and Statistical Manual of Mental Disorders 5 (DSM-5) og som fikk utført audiometri i perioden 1996-1998. Målet var å undersøke, med gullstandarden audiometri og standardisert demensutredning, om redusert hørsel er en uavhengig risikofaktor for demens. I tillegg undersøkte vi om økt demensrisiko også gjaldt for Alzheimer-demens (AD) og andre demenssykdommer enn AD. For å analysere sammenhengene brukte vi Poisson regresjonsanalyse med justering for konfunderende variabler. Studien er forhåndspublisert på ClinicalTrials.gov (NCT04284384).^1^

**Resultater** Ved baseline hadde 1058 (15%) personer redusert hørsel med en høreterskel på 25 decibel (dB), og ved oppfølging hadde 1089 (15%) demens. I den totale gruppen hadde personer med redusert hørsel en relativ risiko (RR) på 1,04 (95% konfidensintervall (KI) 1,00-1,09) per 10 dB økning i høreterskel. For personer under 85 år på oppfølgingstidspunktet var RR 1,12 (95% KI 1,05-1,21). Resultatene for AD og andre demenssykdommer var sammenlignbare. Det var ingen sammenheng mellom redusert hørsel og demens for personer 85 år eller eldre.

**Tolkning** Vi fant en moderat sammenheng mellom objektivt målt hørselssvekkelse og demens i den yngste aldersgruppen (<85 år). Funnene med ingen assosiasjoner for personer i den eldste aldersgruppen (≥85 år) er trolig grunnet død som konkurrerende risiko. Denne studiens bidrag til litteraturen er å vise at ervervet hørselssvekkelse er en risiko for demens over en tidsperiode som er for lang til å kunne forklares med revers kausalitet, og med pålitelig korrigering for konfunderende variabler. Videre forskning er nødvendig for å utforske sammenhengen mellom de forskjellige etiologiene bak redusert hørsel og ulike demensformer, og forskjeller i risiko mellom kjønnene.

**Finansiering** Nasjonalt senter for aldring og helse med støtte fra Helse Sør-Øst RHF (2019024).

**Nøkkelord** Demens, Alzheimers sykdom, redusert hørsel, observasjonsstudie, kohortstudie

1. Myrstad C. Hearing Impairment as a Risk Factor for Dementia in Older Adults. 2021-09-14 2020. <https://classic.clinicaltrials.gov/ct2/show/NCT04284384> (accessed 2023-10-13 2023).
